# HIV-1 capsid undergoes coupled binding and isomerization by the nuclear pore protein NUP358

**DOI:** 10.1186/1742-4690-10-81

**Published:** 2013-07-31

**Authors:** Katsiaryna Bichel, Amanda J Price, Torsten Schaller, Greg J Towers, Stefan MV Freund, Leo C James

**Affiliations:** 1 Protein and Nucleic Acid Chemistry Division, Medical Research Council Laboratory of Molecular Biology, Hills Road, Cambridge, CB2 0QH, UK; 2 Division of Infection and Immunity, University College London Medical Research Council Centre for Medical Molecular Virology, 90 Gower St, London, WC1E 6BT, UK; 3 Current address: Department of Infectious Diseases, King’s College London Guy’s Hospital, Great Maze Pond, London, SE1 9RT, UK

**Keywords:** HIV-1, Cyclophilin, NUP358, Isomerization, Nuclear pore

## Abstract

**Background:**

Lentiviruses such as HIV-1 can be distinguished from other retroviruses by the cyclophilin A-binding loop in their capsid and their ability to infect non-dividing cells. Infection of non-dividing cells requires transport through the nuclear pore but how this is mediated is unknown.

**Results:**

Here we present the crystal structure of the N-terminal capsid domain of HIV-1 in complex with the cyclophilin domain of nuclear pore protein NUP358. The structure reveals that HIV-1 is positioned to allow single-bond resonance stabilisation of exposed capsid residue P90. NMR exchange experiments demonstrate that NUP358 is an active isomerase, which efficiently catalyzes *cis-trans* isomerization of the HIV-1 capsid. In contrast, the distantly related feline lentivirus FIV can bind NUP358 but is neither isomerized by it nor requires it for infection.

**Conclusion:**

Isomerization by NUP358 may be preserved by HIV-1 to target the nuclear pore and synchronize nuclear entry with capsid uncoating.

## Background

Lentiviruses are unique amongst retroviruses in that they can infect non-dividing cells [1]. This attribute is particularly important in the pathogenesis of HIV-1, which can infect macrophages and CD4+ T cells and establish latency [2]. Other retroviruses, such as the gammaretrovirus Murine Leukemia Virus (MLV), require mitosis for integration and productive infection [3]. How lentiviruses infect non-dividing cells and why gammaretroviruses do not is unknown. The ability of lentiviruses to infect non-dividing cells is thought to require use of the nuclear pore [4]. Depletion of NUP358 (also known as RanBP2), a core component of the nuclear pore, reduces HIV-1 infectivity, 2-LTR circle formation and proviral integration and leads to integration site mis-targeting [5-8]. Importantly, substitution of HIV-1 capsid with capsid from the gammaretrovirus MLV results in integration site targeting that phenocopies the effect of NUP358 depletion [5].

The lentiviral capsid protein differs from its gammaretrovirus counterpart in that it has an extra loop between helices 4 and 5 of its N-terminal domain (CA^N^), called the CypA-binding loop. The presence of this loop is conserved in all lentiviruses, despite their considerable divergence and continuous rapid evolution. The cyclophilin A-binding loop is so-called because it is the site of interaction with the cellular isomerase, cyclophilin A (CypA). CypA is a *cis-trans* prolyl isomerase, which catalyzes bidirectional isomerization of *cis* and *trans* forms of proline. Elegant NMR ZZ-exchange experiments by Bosco *et al.* have demonstrated that the lentiviral capsid is a substrate for CypA, suggesting that CypA may catalyze viral uncoating by isomerization of the capsid G89-P90 peptide bond [9]. A specific role for isomerization has never been proven, largely as it has not been possible to mutate either the virus capsid or CypA in order to preserve binding but abolish catalysis. However, the CypA-binding loop is essential for viral infectivity, as mutation of CypA-binding loop residues G89 or P90 in HIV-1 capsid prevent efficient replication [6,10,11].

CypA is potently inhibited by cyclosporine A (Cs), which binds with an affinity of ~1nM [12]. However, the effect of Cs addition on infectivity is highly variable and is not sufficient to render virus non-infectious [10,13,14]. Furthermore, deletion of the CypA gene in CD4+ T cells does not prevent HIV-1 replication [15]. This suggests that whilst the CypA-binding loop is indispensible, CypA binding is not. Moreover, whilst all lentiviruses have a CypA-binding loop, they do not all bind CypA or are affected by Cs inhibition [10,16-20]. For instance, HIV-2 does not package CypA or require it for efficient replication [18].

Recent studies have shown that the nuclear pore protein NUP358 contains a C-terminal cyclophilin domain (NUP358Cyp) that interacts with the capsid of HIV-1 [6,21]. Here we show that the capsid of HIV-1 targets NUP358Cyp using its CypA-binding loop and the mechanistic consequences of this interaction. The crystal structure of the complex between NUP358Cyp and the N-terminal domain of HIV-1 reveals how interaction is maintained despite significant variation between NUP358Cyp and CypA. NMR exchange spectroscopy experiments demonstrate that HIV-1 capsid is a substrate for NUP358Cyp, which catalyzes its isomerization more efficiently than CypA. Finally, exchange experiments on both uniformly and selectively labeled FIV capsid show that FIV is bound but not isomerized by NUP358Cyp and this lack of isomerization correlates with the inability of FIV to use NUP358 as a co-factor. Together the data suggests that two of the defining features of lentiviruses – the CypA binding loop and the ability to infect nondividing cells – are connected through NUP358. One function of the conserved CypA-binding loop may therefore be to mediate interaction with NUP358 and the nuclear pore. Furthermore, interaction with NUP358 may allow HIV-1 to couple capsid uncoating with nuclear entry for efficient infection.

## Results

**Figure 1:**
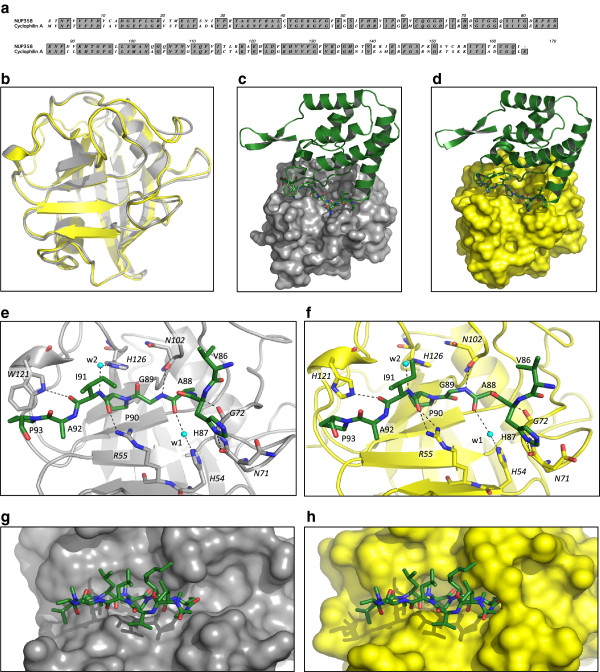
**HIV-1 CA**^**N**^**:NUP358Cyp complex. (a)** Sequence alignment of NUP358Cyp with CypA (conserved regions are shaded and in bold). **(b)** Structural alignment of NUP358Cyp (yellow) from our structure with CypA (gray) from the HIV-1 CA^N^:CypA structure (pdb 1AK4 [23]. **(c)** HIV-1 CA^N^ (green) bound to CypA (molecular surface; gray) (pdb 1AK4 [23]). **(d)** HIV-1 CA^N^ (green) bound to NUP358Cyp (molecular surface; yellow). **(e)** Detailed view of interactions between the CypA-binding loop of HIV-1 CA^N^ (green ball-and-stick) and CypA (gray). **(f)** Detailed view of interactions between the CypA-binding loop of HIV-1 CA^N^ (green ball-and-stick) and NUP358Cyp (yellow). NUP358Cyp residues are numbered according to the equivalent CypA numbering. **(g-h)** Structure of CypA:Cs complex (2RMA [24]) **(g)** or model where NUP358Cyp has been substituted for CypA **(h)**. Cs is in green.

**Figure 2:**
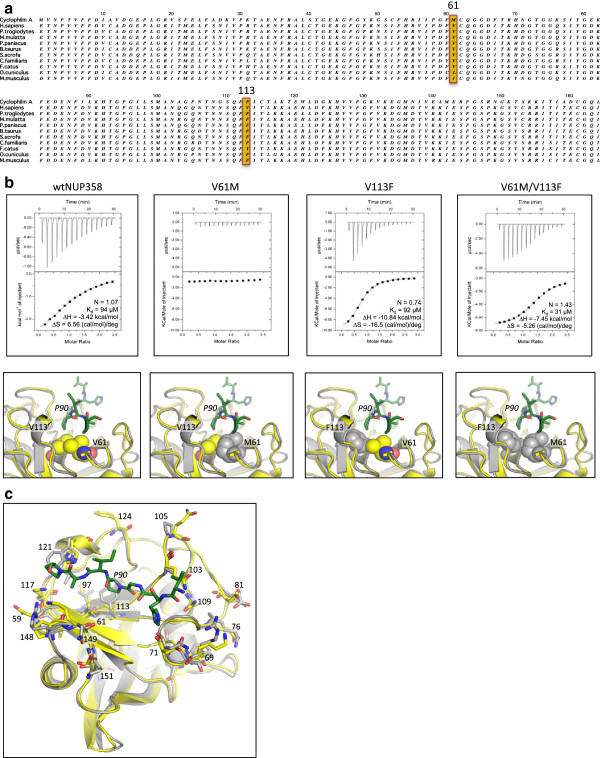
**Co-operative NUP358 residues determine HIV-1 capsid binding. (a)** Sequence alignment of NUP358Cyp from primates and other mammals. Residues at position 61 and 113 are highlighted. **(b)** ITC traces for HIV-1 CA^N^ binding to NUP358Cyp mutants. The location of mutated residues are indicated in the figures below each trace, with residues 61 and 113 shown as space-filling spheres. CypA is indicated in gray and NUP358Cyp in yellow. **(c)** Structural alignment of CypA (gray) from HIV-1 CA^N^:CypA complex 1AK4 with HIV-1 CA^N^:NUP358Cyp (yellow). The CypA-binding loop from HIV-1 CA^N^ is shown in green. Divergent residues in proximity to the binding site are indicated.

The cyclophilin domain from human NUP358 (NUP358Cyp) shares only 65% amino acid sequence identity with CypA (Figure 1a). To determine how NUP358Cyp binds HIV-1 we solved the X-ray crystal structure of HIV-1 CA^N^ in complex with NUP358Cyp at 1.95 Å resolution (see Additional file 1 for data collection and refinement statistics). As observed for the recently published structure of uncomplexed NUP358Cyp [21], despite the sequence variation between CypA and NUP358Cyp, the structures of the two cyclophilins are remarkably similar (Cα atoms show an r.m.s.d of 0.5 Å, Figure 1b). The slot-like binding site found in CypA is preserved in NUP358Cyp and the complex of HIV-1 CA^N^ with NUP358Cyp has a similar quaternary arrangement to the CypA complex (Figure 1c,d). The CypA-binding loop of HIV-1 CA^N^ projects down into NUP358Cyp and mediates almost all direct interactions. In comparison to CypA, NUP358Cyp has residues with bulkier side-chains at the capsid interface, such as K117 and D59 (A117 and G59 in CypA), resulting in a more extended active site surface. This is reflected in a larger buried surface area in the NUP358Cyp:HIV-1 CA^N^ complex (610 Å) compared to CypA:HIV-1 CA^N^ (420 Å). This increased surface area is consistent with the larger entropic change (and presumed greater solvent release) associated with NUP358Cyp:HIV-1 CA^N^ versus CypA:HIV-1 CA^N^ binding (Figure 2b) [5,6,22]. Nevertheless, almost all of the interactions in the NUP358Cyp complex are physico-chemically analogous with CypA:HIV-1 interactions. For instance, the carbonyl of I91 in CA^N^ makes an important hydrogen bond interaction with CypA via the pyrrole ring of W121 and with NUP358Cyp via the imidazole side chain of H121 (Figure 1e,f). Sequence variation between NUP358Cyp and CypA is also accommodated because some interactions with HIV-1 CA^N^ are made via main-chain atoms. An important hydrogen bond interaction in both NUP358Cyp and CypA occurs between the side-chain of CA^N^ H87 and the peptide oxygen of residue 71 in the cyclophilins (Figure 1e,f). Despite the physico-chemical similarities between NUP358Cyp and CypA, there are functionally relevant structural differences. Cs inhibits capsid binding to CypA but not NUP358Cyp [6]. The large cyclic peptide drug is accommodated within the active site of CypA but superposition of the CypA-Cs complex on NUP358Cyp reveals that steric clashes preclude binding of Cs to NUP358Cyp (Figure 1g,h).

Subsequent to our previously published binding data between HIV-1 CA^N^ and NUP358Cyp [6], we identified conditions that allowed higher concentrations of NUP358Cyp to be achieved. This allowed us to repeat the ITC binding between HIV-1 CA^N^ and NUP358Cyp using significantly higher protein concentrations than previously. This gave binding data with a much stronger signal and consequently a more accurate fit, resulting in a revised Kd of 94 μM (Figure 2b). This is similar to the affinity measured by Lin *et al.* for the same interaction (Kd ~ 200 μM) [21].

Previous analysis of codon-specific selective pressures has revealed that NUP358Cyp is under positive selection, consistent with its role as a viral host co-factor [6]. In particular, it was found that residue 61 has diverged from ancestral CypA such that it no longer encodes a methionine but rather a valine, leucine or isoleucine (Figure 2a). The importance of residue 61 was demonstrated by reversing this change in human NUP358Cyp (V61M), which was sufficient to abolish interaction with HIV-1 in a cellular TRIM-NUP358 assay [6]. Analysis of the HIV-1 CA^N^:NUP358Cyp structure reveals that residue 61 is located at the center of the binding site, almost directly under CA^N^ P90 (Figure 2b). However, the presence of a methionine residue at this position is not itself sufficient to prevent HIV-1 CA^N^ binding, as this residue is accommodated in HIV-1 CA^N^:CypA complexes [25]. Examination of the side-chains surrounding position 61 reveals that it is within 4 Å (C_β_-C_β_) of residue 113, which uniquely in humans is a valine, having diverged from the ancestral phenylalanine found in CypA and other species’ NUP358Cyp (Figure 2a). The residue at position 113 has been shown to be important for the interaction of both CypA and NUP358Cyp with HIV-1 CA^N^, as mutation of this residue to the bulky aromatic residue tryptophan in either cyclophilin prevents binding to HIV-1 CA^N^[21,26]. To determine whether these two residues, 61 and 113, are acting cooperatively to dictate HIV-1 CA^N^ binding, we made combinations of these mutations on the background of NUP358Cyp and tested their binding to HIV-1 CA^N^ by ITC (Figure 2b). In agreement with the published TRIM-NUP358 assay [6], we found that V61M was sufficient to abolish interaction with HIV-1 CA^N^. In contrast, mutation V113F preserves HIV-1 binding. Moreover, when the V61M mutation is made together with V113F (V61M/V113F) then binding is also preserved. Thus V61M abolishes binding of HIV-1 to NUP358Cyp because a methionine at position 61 is tolerated when position 113 is a phenylalanine (as in CypA) but not when it is a valine (as in NUP358Cyp). The reason for this sensitivity to particular combinations at positions 61 and 113 is likely due to the fact that these residues form the core of the hydrophobic pocket into which CA^N^ P90 binds (Figure 2c). Intriguingly, only in humans has NUP358Cyp acquired the F113V mutation (with respect to ancestral CypA). In other primate species, canines and rodents, F113 is conserved. Mutation M61V pre-dates F113V, but had human NUP358Cyp acquired its F113V mutation on an ancestral M61 background then HIV-1 would not be able to bind NUP358Cyp in human cells.

As HIV-1 capsid is not the natural ligand for NUP358Cyp, we used the complexed structure to assess the specificity of the interaction. If there were selection pressure on the virus to maintain the interaction we would expect a higher degree of shape complementarity than if it were serendipitous cross-reaction. Using the CCP4 program ‘SC’, we calculated a surface complementarity score (Sc) for the HIV-1 CA^N^:NUP358Cyp complex of 0.78. As typical protein:protein interactions have Sc scores from 0.6-0.8[27], this indicates that the interaction is highly specific. Calculation of Sc scores from HIV-1 CA^N^:CypA complexes gives similar scores (~0.8). Whilst the sequence differences between NUP358Cyp and CypA have not significantly altered its conformation, NUP358Cyp does have altered surface electrostatics (Additional file 2). NUP358Cyp differences with respect to CypA in and around the active site such as K76Q and R148P mean that NUP358Cyp has a negatively charged surface at physiological pH (pI of 5.9) compared to CypA, which is positively charged (pI of 7.7). However, the positively charged key catalytic residue R55 is conserved in both molecules and adopts a comparable position in their active sites (Figure 1e,f). The conservation of R55 positioning and its proximity to CA^N^ P90 suggested to us that R55 may hydrogen bond with the peptide oxygen of P90 and lower the activation energy for *cis-trans* prolyl isomerization.

**Figure 3:**
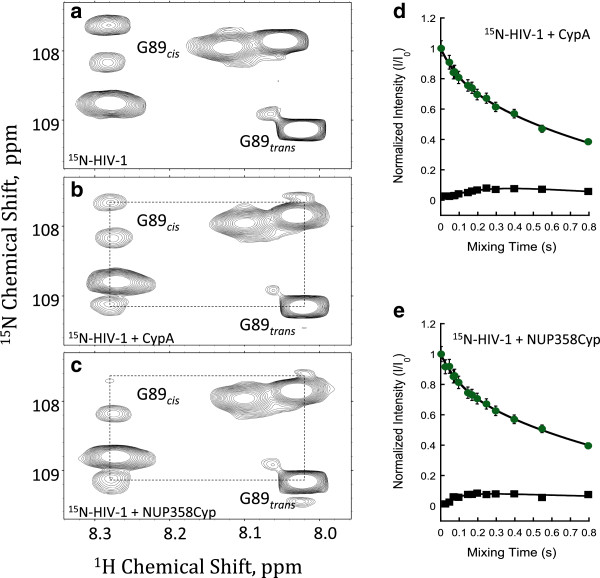
**NUP358Cyp catalyses *cis-trans *isomerization of HIV-1 CA**^**N**^**. (a**-**c)** 2D ^1^H-^15^N ZZ-exchange spectra of HIV-1 CA^N^ focused on Gly89, without enzyme **(a)**, in the presence of NUP358Cyp **(b)** or CypA **(c)**. Gly89 *cis,trans*^1^H,^15^N correlation or “auto” peaks are labeled and are the result of *cis* and *trans* forms of the proceeding Pro90 residue being populated at equilibrium. Addition of either NUP358Cyp or CypA yields exchange peaks that connect *cis* and *trans* auto peaks (broken lines). The *cis* exchange peak appears at the same ^1^H (^15^N) chemical shift position as the *trans* auto peak and vice versa. Note, that the *trans* exchange peak is largely obscured by additional signals in the spectra. **(d**-**e)** The intensities of both auto and exchange peaks vary as a result of the ZZ-mixing time (T_m_). Fits of CypA **(d)** and NUP358Cyp **(e)** yield exchange constants of 4.3 and 12.1 s^-1^ respectively.

The cyclophilin domain of NUP358 has been shown to be an active isomerase, although it possesses much weaker catalytic activity than CypA when tested using a synthetic proline-containing peptide substrate [21]. So far, the only biological role assigned for NUP358Cyp has been to facilitate the interconversion of thermodynamically or kinetically trapped isoforms of red/green opsin in cone cells via *cis-trans* prolyl isomerization of proline residues within opsin [28]. Given that CypA has been shown to isomerize HIV-1 capsid [9], we investigated whether CA^N^ is also a substrate for NUP358Cyp using NMR ZZ-exchange spectroscopy. Previous work by Bosco *et al.* and Eisenmesser *et al.* has shown that ZZ-exchange is an effective way of measuring CypA isomerization under steady state conditions [9,29]. 2D ^1^H-^15^N ZZ-exchange data on uniformly ^15^N-labeled CA^N^ were collected in the presence of NUP358Cyp and CypA and compared to intrinsic ZZ-exchange of ^15^N-labeled CA^N^ alone. ^1^H-^15^N correlation spectra require amide protons and therefore proline residues (such as P90) are not detected. However, the adjacent residue, G89 is characterised by two ^1^H-^15^N correlation peaks indicating that the proceeding proline must exist in both *cis* and *trans* forms (Figure 3a-c). These “auto” peaks are detected in the absence or presence of NUP358Cyp or CypA, respectively. An estimation of the *cis/trans* distribution at equilibrium using ^1^H-^15^N correlation (HSQC) spectra revealed that ~14% of the capsid G89-P90 bond exists in *cis* and ~86% *trans*, as previously observed [9]. The introduction of a variable mixing period in ZZ-exchange experiments enables inter-conversion between the *cis* and *trans* isomers resulting in the mixing time dependent build up of “exchange” peaks which connect “auto” peaks in a distinct pattern. For CA^N^ alone, there is no detectable magnetization transfer between the two species, as evidenced by the lack of “exchange” peaks at all time points. This suggests that uncatalyzed *cis-trans* isomerization is very slow, with an exchange rate < 0.1 s^-1^. However, addition of catalytic amounts (1:10 molar ratio) of either CypA or NUP358Cyp results in the rapid build-up of intense exchange peaks, indicating fast *cis-trans* isomerization is now taking place (Figure 3b,c). These experiments unambiguously confirm HIV-1 capsid as a substrate for NUP358Cyp isomerization.

In order to determine the efficiency of NUP358Cyp isomerization of HIV-1 capsid and compare this to CypA, we analysed mixing time dependent “exchange” and “auto” peak intensities, as previously described [9]. We found that CypA catalyzed capsid isomerization with a rate of 4.3 ± 2.3 s^-1^ (Figure 3d) (similar to previously described values of 6.6 ± 2.4 [30]). Under similar conditions, NUP358Cyp catalyzed HIV-1 isomerization between 2–3 times faster than CypA, at a rate of 12.1 ± 3.4 s^-1^ (Figure 3e). This data suggests that, despite the weaker interaction, HIV-1 capsid might be a better substrate for isomerization by NUP358Cyp than by CypA. Capsid isomerization has been hypothesized to act as a stimulus for viral uncoating [9,31]; therefore it is possible that an increase in the rate of capsid isomerization due to NUP358Cyp might act as a trigger for HIV-1 uncoating and allow nuclear entry.

**Figure 4:**
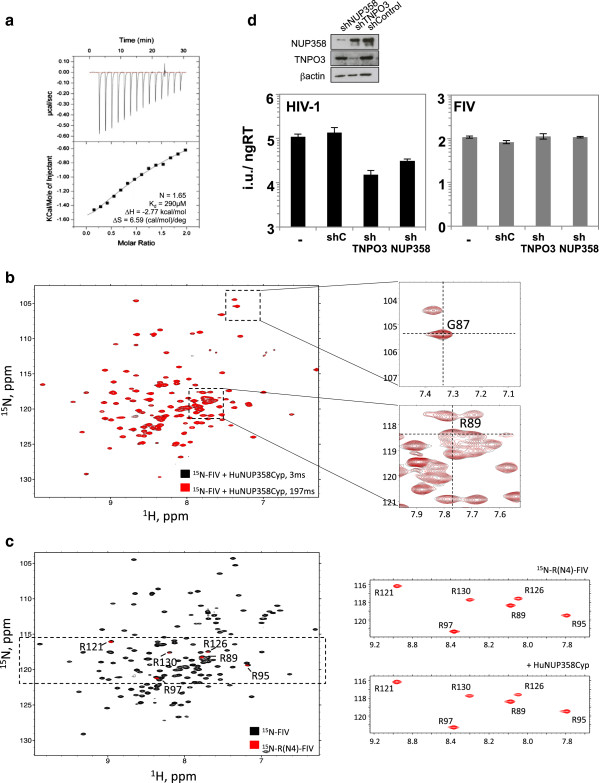
**FIV uncouples binding and isomerization by NUP358. (a)** ITC trace of NUP358Cyp interaction with FIV CA^N^. **(b)** Overlay of 2D ^1^H-^15^N ZZ-exchange spectra of FIV CA^N^ in the presence of NUP358Cyp at 3 and 197 ms mixing times. Zoomed-in views of the peaks corresponding to G87 and R89 are also shown. The cross-hairs indicate where exchange peaks would be situated, if *cis-trans* isomerization took place. **(c)** Overlay of uniformly ^15^N-labeled and selective ^15^N-Arg labeled FIV CA^N^. Spectra of selective ^15^N-Arg labeled FIV CA^N^ are also shown in panels on the right in the absence (top) and presence (bottom) of human NUP358Cyp. **(d)** Upper: Western blots showing depletion of NUP358 or TNPO3 in HeLa cells using antibodies against NUP358, TNPO3 or β-Actin as loading control [6]. Lower: VSV-G pseudotyped GFP-encoding HIV-1 or FIV infection of HeLa cells and HeLa cells expressing scrambled control (shC) or NUP358 or TNPO3 specific shRNA [6,33] (mean of three independent viral doses, error bars show standard deviation). Titers are plotted as infectious units per ng of reverse transcriptase activity. Data are representative of two independent experiments.

Finally, we attempted to address whether NUP358Cyp-mediated isomerization is important for viral infection. Despite extensive study, no HIV-1 mutants have been found which bind NUP358 or CypA but are not isomerized. Therefore, we decided to investigate the capsid of a naturally occurring divergent lentivirus. The CypA-binding loop is present in all lentiviruses but the sequence varies extensively, particularly between primate and feline lineages. To determine whether there is any separation of binding and isomerization in the feline lineage we extended our study to look at feline immunodeficiency virus (FIV). FIV can bind to CypA [32] but has a very different loop sequence to HIV-1, with an ‘RP’ motif at positions 89–90 rather than a ‘GP’ motif. Nevertheless, as for HIV-1, P90 has been shown to be an important residue for interaction of FIV CA^N^ with CypA [32]. Testing by ITC revealed that FIV also binds human NUP358Cyp (Figure 4a). Next we examined ^15^N-labeled FIV CA^N^ to establish whether it is isomerized by human NUP358Cyp. Unlike with HIV-1, addition of catalytic amounts of NUP358Cyp did not lead to any significant peak changes in variable mixing period ZZ-exchange experiments, suggesting that FIV is not isomerized by human NUP358Cyp (Figure 4b). The FIV spectrum around R89 is densely populated, so to rule out the possibility that isomerization peaks are masked, we expressed and purified CA^N^ selectively labeled with ^15^N arginine. There are six arginine residues in FIV CA^N^ and six peaks are observed in the HSQC spectra from the selectively labeled protein (Figure 4c). Furthermore, these peaks overlay well with the equivalent peaks from the uniformly labeled protein. With this system, we re-examined ZZ-exchange upon addition of human NUP358Cyp. As can be clearly seen, there is no change in any of the arginine residues, including R89 (Figure 4c). Taken together, the data show that FIV is bound but not isomerized by human NUP358. Finally, we compared infection of HIV-1 and FIV attenuated GFP virus in HeLa cells stably transduced with either empty vector or vector encoding shRNA against NUP358 or TNPO3 (Figure 4d). As previously shown, depletion of NUP358 or TNPO3 inhibits infection of HIV-1 [6-8,33,34]. However, depletion of these proteins has no effect on FIV infection (Figure 4d) [34,35]. It is noteworthy that, in FIV, loss of NUP358 isomerization correlates with lack of dependence on this co-factor for infection. Moreover FIV infection also occurs independently of TNPO3 (Figure 4d) [34,35], a nuclear transport factor that is involved in the same HIV-1 nuclear entry pathway as NUP358 [6,34] and whose subcellular localization has been shown to be affected by NUP358 depletion [36].

## Discussion

Nuclear pore protein NUP358 was identified as a candidate co-factor for HIV-1 replication in two independent siRNA screens [37-39]. Depletion of NUP358 has subsequently been shown to inhibit HIV-1 replication and interfere with targeting viral integration into the host genome [5-8]. Here we have provided a structural basis for HIV-1 CA^N^:NUP358Cyp interaction. Furthermore, we have shown that NUP358Cyp interaction, but not isomerization, is conserved by FIV, a virus that does not require NUP358 for infection. This is significant as it provides the first distinction between binding and isomerization of a lentiviral capsid by a cyclophilin, and may shed light on the role of NUP358 interaction in lentiviral infection.

Recent microscopy data have shown that intact HIV-1 capsid cores can dock at the nuclear pore [40] and that this perinuclear localization is dependent on NUP358 [8]. NUP358 is a large, multidomain protein that forms the cytoplasmic filaments of the vertebrate nuclear pore complex [41,42]. There are eight copies of NUP358 at the nuclear pore, which form a ring of ~35-50 nm filaments on the cytoplasmic side of the nuclear pore and project into the cytoplasm [43,44]. NUP358 contains four RanGTP binding domains, two Zinc fingers, several FG repeats and a C-terminal cyclophilin domain [41,45]. The C-terminal cyclophilin domain, which is bound by HIV-1 CA^N^, is found at the tips of the cytoplasmic filaments, as evidenced by the fact that deletion of this domain still allows NUP358 to localize to the nuclear membrane, and expression of this domain on its own is not sufficient to localize to the nuclear membrane [8,21]. The direct interaction we observe between NUP358Cyp and the HIV-1 capsid provides one explanation for how HIV-1 cores dock at the nuclear pore, without precluding the possibility that other domains of NUP358 might cooperate to facilitate nuclear docking, perhaps via non-specific interactions of HIV-1 cores with FG repeats [8]. Use of the CypA-binding loop by HIV-1 to target the virus to the nuclear pore via interaction with the cyclophilin domain of NUP358 also provides an explanation for the key observation that substitution of HIV-1 CA with MLV CA prevents HIV-1 infection of non-dividing cells [46].

NUP358 plays an important role in nucleocytoplasmic transport, being the site of interaction with Ran (the small GTPase that regulates nucleocytoplasmic transport by proteins of the karyopherin β family [47]), SUMO-modified RanGAP (the mammalian RanGTPase-activating protein that is highly concentrated at the cytoplasmic side of the nuclear pore complex [48,49]), and the export receptor CRM1 [50]. Nup358 has also been suggested to act as a platform to recruit import receptors to pre-bound cargos, as cellular substrates DBC-1 and DMAP-1 have been shown to directly bind to NUP358 and be transported by importin α/β [51]. Therefore, recruiting to the nuclear pore via CA interaction with NUP358 may allow HIV-1 access to importin or transportin routes into the nucleus and drive integration into actively transcribing regions of the genome.

The presence of intact capsid at the nuclear pore has led to the suggestion that capsid uncoating may take place after pore docking. This hypothesis is attractive, as last-minute uncoating provides several potential advantages for the virus. First, it allows the viral proteins, enzymes and nucleic acids to be kept together – increasing enzymatic efficiency and ensuring that components are at the right place at the right time. Second, late uncoating means that the virus genome is protected within its capsid for as long as possible from the cytosol of the host cell. The host cytosol is a dangerous place for the virus genome, as it contains pattern recognition receptors such as the RIG-I-like receptors and AIM-2-like receptors which recognize cytoplasmic viral nucleic acid [52] and restriction factors such as APOBEC3G [53]. However, by maintaining an intact capsid inside the cell, the virus becomes vulnerable to innate capsid-targeting restriction factors such as TRIM5α and, in certain primate species, TRIMCyp [22,54-56]. A problem with the late uncoating model is the question of how uncoating is triggered. Elegant experiments using cyclosporine inhibition of TRIMCyp restriction suggest that uncoating may be facilitated by reverse transcription, since inhibition of reverse transcription delays the process of uncoating [57]. The rate of this process may be linked to the time required for virus to recruit to the pore, ensuring that uncoating occurs immediately prior to nuclear entry. Alternatively, it has been suggested that isomerization of capsid by CypA may be the trigger for viral uncoating [9], but if this is the case then uncoating would be expected to occur as soon as the capsid encounters the cytoplasm upon cell entry. We have shown that NUP358Cyp is an active prolyl isomerase, capable of catalyzing the isomerization of HIV-1 capsid more efficiently than CypA. It is therefore possible that by interacting with NUP358Cyp, HIV-1 can both recruit to the pore and ensure uncoating occurs at the right place at the right time. Unfortunately, without the ability to make mutations in the capsid of HIV-1 that preserve binding but abrogate isomerization it is not possible to directly test this hypothesis. However, we have shown that FIV can be used as a model virus in which binding to human NUP358Cyp occurs but isomerization does not. It is tempting to speculate that the inability of FIV to be isomerized by NUP358Cyp may explain why it does not use NUP358 and associated transportin TNPO3 for nuclear entry and infection in human cells [34,35]. However, further experiments on fully infectious FIV in spreading infection of primary feline cells will be necessary to comprehensively unpick its nuclear import pathway.

On the basis of our crystallographic and NMR data, we speculate that interaction between capsid and NUP358 may allow HIV-1 to recruit to the nuclear pore and synchronize uncoating with nuclear entry. This may help to explain why depletion of NUP358 affects HIV-1 infectivity and integration targeting, why HIV-1 capsids dock at the pore and why all lentiviruses preserve their CypA-binding loops.

## Conclusions

We have solved the crystal structure of HIV-1 CA^N^ in complex with the cyclophilin domain of nuclear pore protein NUP358, revealing how HIV-1 may target itself to the nuclear pore during infection. Accompanying NMR data also reveal that NUP358Cyp isomerizes HIV-1 CA^N^ more efficiently than CypA but is unable to isomerize capsid from the distantly related FIV. This result may be significant because FIV, unlike HIV-1, does not require NUP358 for infection. Our data suggest that isomerization by NUP358 may be preserved by HIV-1 both to target the nuclear pore and synchronize nuclear entry with capsid uncoating.

## Methods

### Protein expression and purification

HIV-1 M-group (NL4-3) N-terminal capsid domain (HIV-1 CA^N^; residues 1–146 (Gag residues 133–278); AAB60571.1) was cloned into a tagless expression vector, and FIV N-terminal capsid domain (FIV CA^N^; residues 1–139 (Gag residues 136–274); AAU12277.1), human CypA (P62937.2) and the cyclophilin domain from human NUP358 (residues 3057–3224; P49792.2) were cloned into an expression vector containing an N-terminal His_6_-tag. All proteins were expressed in *E. coli* C41(DE3) cells, induced with 100 μM IPTG at OD_600_ of 0.8 and incubated overnight at 20°C. Uniformly ^15^N-labeled CA^N^ domains of HIV-1 or FIV were expressed in *E. coli* in K-MOPS buffer supplemented with 20 mM ^15^NH_4_Cl as the sole source of nitrogen. Selectively labeled FIV CA^N^ was prepared by expressing in the presence of ^15^N-arginine (^15^N-R(N4)) or with ^15^N and ^13^C sources. HIV-1 CA^N^ was purified as described [58]. His_6_-tagged proteins were purified using Ni-NTA beads (Qiagen) and gel-filtration chromatography in 75 mM Tris pH 8.0, 50 mM NaCl, 1 mM DTT. All mutant proteins were expressed and purified as per the wild-type proteins.

### Crystallization and structure determination

Crystals of HIV-1 CA^N^ in complex with NUP358Cyp were grown in the following conditions: protein solution (0.55 mM each of HIV-1 CA^N^ and NUP358Cyp in 10 mM potassium phosphate pH 7.4, 1 mM DTT) was mixed with reservoir solution (23% v/v PEG 4000, 23% glycerol, 8.5% isopropanol, 85 mM HEPES pH 7.5, 20 mM spermine tetrahydrochloride, 100 mM glycine) in a 1:1 mix. Drops (total volume 400 nl) were set up in sitting drop format and the plates incubated at 17°C. Crystals grew within 48 h, to a size of 0.14 mm x 0.04 mm x 0.04 mm. Crystals were flash-frozen in liquid nitrogen, and data collected at beamline ID14-1 at the ESRF (Grenoble, France). Data were processed using MOSFLM [59] and the CCP4 suite [60]. All structures were determined by molecular replacement in Phaser [61], using the structure of HIV-1 CA^N^ from 1AK4 [23] as a search model. Model building was performed using Coot [62] and refinement was carried out using REFMAC5 [63]. Model validation was performed using MolProbity [64]. Crystallization and refinement statistics are shown in Additional file 1. Figures were prepared using PyMOL.

### Isothermal titration calorimetry

Samples were dialysed against 50 mM potassium phosphate pH 7.4, 100 mM NaCl, 1 mM DTT. Protein concentrations were determined by absorbance at 280 nm. ITC experiments were carried out using a MicroCal ITC200 calorimeter, with NUP358Cyp (typical concentration 2.5 mM) in the syringe and HIV-1 CA^N^ (typical concentration 0.2 mM) in the cell. Experiments were conducted by titrating capsid (syringe) into cyclophilin (cell) at 15°C. Data were analysed using MicroCal Origin 7.0 implementing a simple one set of binding sites model.

### Protein assignments

The spectra of ^15^N,^13^C-His_6_-FIV CA^N^ and ^15^N,^13^C-HIV-1 CA^N^ were acquired on a Bruker 600 MHz spectrometer at 298 K using a final concentration of 500 μM (FIV) and 330 μM (HIV-1) protein sample. Data processing was performed in TopSpin 3.0 (Bruker, Karlsruhe).

### 2D ^1^H-^15^N Heteronuclear (ZZ) Exchange Spectroscopy

For all NMR experiments, proteins were dialysed against 50 mM potassium phosphate pH 6.5, 1 mM DTT. Capsids were used at 12-fold excess concentration over CypA or NUP358Cyp as previously described [9,30]: 430 μM CA^N^ and 35 μM CypA or NUP358Cyp, or 430 μM CA^N^ only. All NMR samples contained 5% D_2_O. 2D ^1^H-^15^N heteronuclear (ZZ) exchange was performed as previously described [65] using published models [9,30]. The experiments were performed on a Bruker 800 MHz spectrometer at 298 K using an in-house written pulse program with mixing times collected in a randomised order. The first time-point was acquired twice to assess the error. The data was processed in TopSpin 3.0 (Bruker, Karlsruhe) after analysis in Sparky (T. D. Goddard and D. G. Kneller, University of California, San Francisco).

### Infection assays

VSV-G pseudotyped vectors derived from HIV-1 and FIV have been described, as has their preparation by 293 T transfection [66]. Viral doses were measured by reverse transcriptase enzyme linked immunosorbant assay (Roche). Viral vector infection assays using VSV-G pseudotyped viral vectors encoding GFP were analyzed by enumerating the number of green cells 48 hours post infection by flow cytometry. Stable HeLa cell clones expressing NUP358 specific or TNPO3 specific short hairpin RNA (shRNA) from MLV vector pSIREN RetroQ (Clontech) have been described [6]. Scrambled control shRNA (shC) is a mismatch against TNPO3, and is described in [33].

### Protein Data Bank accession number

Coordinates for the HIV-1 CA^N^:NUP358Cyp complex crystal structure have been deposited into the RCSB Protein Data Bank with the accession code 4LQW.

## Abbreviations

HIV-1: Human immunodeficiency virus type 1; CAN: N-terminal capsid domain; FIV: Feline immunodeficiency virus; CypA: Cyclophilin A; Cs: Cyclosporine A; NMR: Nuclear magnetic resonance; HSQC: Heteronuclear single quantum coherence; VSV-G: Vesicular stomatitis virus glycoprotein; shRNA: short hairpin RNA; siRNA: small interfering RNA; ITC: Isothermal titration calorimetry.

## Competing interests

The authors declare that they have no competing interests.

## Authors’ contributions

KB, AJP and TS performed the experiments and analysed data (KB performed the NMR experiments; AJP performed the crystallography and ITC experiments; TS performed the knockdown and infection experiments). GJT, SVF and LCJ conceived of the study and participated in its design. LCJ and AJP wrote the manuscript. All authors read and approved the final manuscript.

## Supplementary Material

Additional file 1 Table of data collection and refinement statistics. Click here for file

Additional file 2 **Surface electrostatics of NUP358Cyp and CypA.** Electrostatic surface potential of NUP358Cyp and CypA, in complex with HIV-1 CA^N^ (yellow sticks) (CypA:HIV-1 CA^N^ pdb 1AK4) as calculated by APBS (Adaptive Poisson-Boltzmann Solver). Blue represents a positive charge and red a negative charge. Scaled from −20 to +20 *kT* e^−1^. Click here for file
