# Supplementary material for: HIV-1 capsid undergoes coupled binding and isomerization by the nuclear pore protein NUP358
Source: Retrovirology. 2013 Jul 31;10:81. doi: 10.1186/1742-4690-10-81 (PMC3750474; doi:10.1186/1742-4690-10-81)
Supplement: Additional file 1 — Table of data collection and refinement statistics. [file 1742-4690-10-81-S1.pdf]

Additional File 1. Data collection and refinement statistics

	NUP358Cyp:HIV-1 CA[N]
**Data collection**	
Space group	*C*2
Cell dimensions	
a, b, c (Å)	142.2, 38.3, 123.8
α, β, γ (°)	90.0, 100.6, 90.0
Resolution (Å)	33 – 1.95
R_{sym}	0.126 (0.448)*
I / σI	7.6 (2.2)
Completeness (%)	94.5 (94.5)
Redundancy	3.2 (3.2)
**Refinement**	
Resolution (Å)	1.95
No. reflections	48,326
R_{work} / R_{free}	0.205/0.243
No. atoms	
Protein	9,862
Ligand/ion	0
Water	380
B-factors	
Protein	17.0
Ligand/ion	n/a
Water	28.7
R.m.s deviations	
Bond lengths (Å)	0.004
Bond angles (°)	0.8

*Values in parentheses are for highest-resolution shell.
