# Supplementary figures and images for: HIV-1 capsid undergoes coupled binding and isomerization by the nuclear pore protein NUP358
Source: Retrovirology. 2013 Jul 31;10:81. doi: 10.1186/1742-4690-10-81 (PMC3750474; doi:10.1186/1742-4690-10-81)

NUP358Cyp

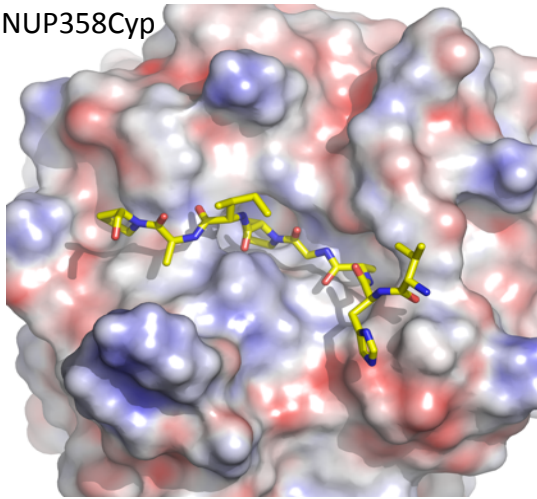

CypA

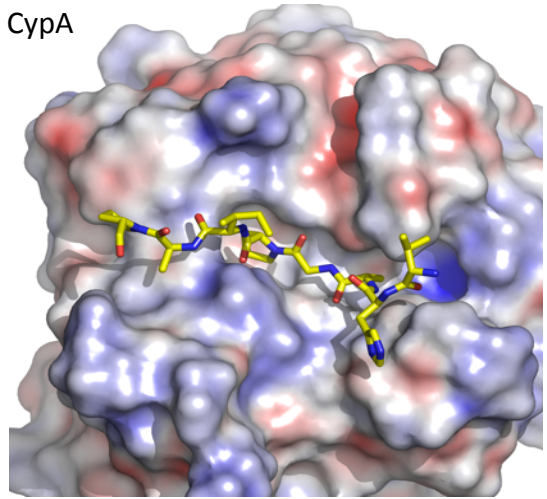

Supplement: Additional file 2 — Surface electrostatics of NUP358Cyp and CypA. Electrostatic surface potential of NUP358Cyp and CypA, in complex with HIV-1 CAN (yellow sticks) (CypA:HIV-1 CAN pdb 1AK4) as calculated by APBS (Adaptive Poisson-Boltzmann Solver). Blue represents a positive charge and red a negative charge. Scaled from −20 to +20 kT e−1. [file 1742-4690-10-81-S2.pdf]
